# Wound Infection after Laparoscopic-Assisted Gastrostomy in Infants

**DOI:** 10.1055/s-0039-1696731

**Published:** 2019-09-04

**Authors:** Linnéa Burman, Maia Diaz, Margrét Brands Viktorsdóttir, Helen Sjövie, Pernilla Stenström, Martin Salö, Einar Ólafur Arnbjörnsson

**Affiliations:** 1 Department of Clinical Sciences Lund, Skåne University Hospital, Lund University, Pediatric Surgery, Lund, Sweden

**Keywords:** laparoscopic-assisted gastrostomy, infants, wound infections, complications, bacterial culture, antibiotics

## Abstract

**Background** Gastrostomy placement in children is one of the most frequently performed pediatric surgical procedures and laparoscopic-assisted gastrostomy (LAG) is the preferred technique. Wound infection after LAG has become a significant concern due to the emergence of antibiotic resistance. The aim of this study was to describe the frequency of wound infection after LAG in children younger than 2 years of age and to identify the associated risk factors and the bacterial species involved.

**Methods** Information about wound infection, results from bacterial cultures, and type of antibiotic treatment used within 30 postoperative days after LAG were compiled for infants who underwent LAG from 2010 to 2017. A retrospective chart review was performed. Data was compiled from charts and from an electronic database containing prospectively collected data. A multivariate logistic analysis was used to explore potential risk factors. Preoperative antibiotic prophylaxis and postoperative local wound care were conducted according to standard procedures.

**Results** The 141 included infants underwent surgery at a median age of 10 months (range: 1–24). Thirty-eight (27%) patients had a clinically determined wound infection, bacteria were cultured from 26/38 (69%), and 30/38 (79%) received antibiotic treatment. The median interval from surgery to detection of a clinical wound infection was 14 days (range: 4–30). The most common microbes discovered were skin bacteria *Staphylococcus aureus* or *Streptococcus pyogenes* , but respiratory and intestinal bacteria were also found. Multivariate logistic regression analysis revealed no independent risk factors for infection such as age, gender, or underlying diagnosis.

**Conclusion** Infants have a high rate of postoperative clinical wound infection after LAG despite the use of preoperative antibiotic prophylaxis and intense local wound care. Gender, age at operation, and previous diagnoses were not found to be independent risk factors for wound infection.

Gastrostomy tube insertion is indicated when patients have insufficient nutritional or medicine intake for a period of > 2 to 3 weeks, 1 or when the need for additional enteral feeding (e.g., through a nasogastric tube) is expected to exceed 3 months. 2

Laparoscopic-assisted gastrostomy (LAG) is the method of choice for children at many centers. 3 4 5 6 7 8 9 10 11 12 Wound infection around the gastrostomy insertion site sometimes occurs after LAG. However, studies on wound infection after gastrostomy procedures in children are scarce and detailed information is unavailable. 13 14 15 16 Knowledge of wound infection and how they can be prevented, identified, or treated is of urgent concern due to the growing worldwide antibiotic resistance.

The aim of this study was to determine the frequency of infections within 30 postoperative days in children up to 2 years of age, the associated risk factors, types of bacteria found at the infection site, and the current use of antibiotics. The rationale for this study was the collection of important information for further improvement of clinical procedures and for the parents. The conceptual model is not well developed and the gaps in understanding are attributable to lack of information regarding the pediatric population. The possible contributors to wound infection in this population are concomitant diseases as well as the corpus alien, specifically the gastrostomy button, in the new gastrostomy wound. The immune status is hampered by the fact that the children are very sick, as well as the antibiotic dose and timing. Thus, the hypothesis is that LAG placement would be associated with the risk factors for postoperative wound infections.

## Materials and Methods

### Surgical Procedures, Perioperative Routines, Postoperative Care, and Follow-Up

The only surgical method used during the study period was the LAG procedure with the U-stitch technique. 2 3 4 The surgical techniques used to perform laparoscopic gastrostomy (LG) tube placement entailed the laparoscopic-assisted placement of a Mickey gastrostomy button (provided by Halyard Health, Inc. [Alpharetta, Georgia] ). Through a lower umbilical skin incision, a mini laparotomy was performed. A 3 to 5 mm VersaStep trocar was safely inserted into the abdomen and pneumoperitoneum was established with CO _2_ insufflation. Using a 3 to 5 mm 30° laparoscopy optic, the stomach was identified. A skin incision was made between the left costal margin and the umbilicus, destined for the gastrostomy. A 5-mm trocar was inserted at this point through the rectus muscle and into the abdominal cavity under visual control with the laparoscope. Through this port, the anterior stomach wall was grasped using an instrument, with clear margins from the pylorus, and exteriorized when the grasper and trocar were pulled back. The stomach was then sutured to the rectus muscle fascia using a continuous double U stitch and by performing a purse string suture around the gastrostomy opening on the stomach wall. The gastrostomy tube was inserted into the cavity of the stomach through a small incision in the stomach wall. Next, the placement of the gastrostomy tube was controlled using a gastroscope at the end of the surgical procedure.

The mean duration of the operative intervention was 45 minutes, including the placement of the gastrostomy and the endoscopy of the stomach. Preoperative antibiotic prophylaxis with intravenously administered trimethoprim/sulfamethoxazole (Eusaprim), according to the child's age, was routinely used within 30 minutes before the start of the operative intervention. 4 The postoperative routines were consistent throughout the study period. The inserted gastrostomy button was connected to the nutrition catheter, which was fixed by tape to the abdominal wall during the first 5 postoperative days, to reduce pain, granuloma formation, and infection by minimizing movement of the button. The skin around the gastrostomy was washed with Hibiscrub twice daily during the first 5 postoperative days. A wound culture was obtained any time considered necessary and treatment with antibiotics was prescribed when considered indicated, which was at the surgeon's discretion. The patients were cared for on the basis of an established protocol including follow-up counseling at one and 3 months postoperatively. The infants were monitored by their caregivers, who adhered to the protocol during their stay at the hospital. After leaving the hospital, protocol deviations may be unavoidable.

### Data Collection

The study was a retrospective study based on a local prospective register for gastrostomy as well as on patients' electronic medical carts. All patients (0 to 2 years old) who underwent the LAG procedure at our tertiary center for pediatric surgery from 2010 through 2017 were included. The collected data consisted of gender, age at operation, underlying diagnoses, weight and length, clinical wound infection, the results of bacterial cultures, and the use of antibiotics within 30 postoperative days, which was the endpoint of the study. Exclusion criteria were missing information in the files.

A 30-day infectious complication classification was used according to Clavien-Dindo grade 2, which assumes that infections presenting within 30 postoperative days are caused by the operation. 17 The criterion for the presence of wound infection was a clinical diagnosis of wound infection, determined by the surgeon.

### Statistics

To evaluate possible risk factors for postoperative wound infection, multivariable regression analysis was conducted, with the 30-day postoperative infection as the outcome, and gender, age at operation, weight, and underlying diagnosis as independent variables. A *p* -value < 0.05 was considered significant. Statistical analysis was performed using IBM SPSS Statistics, version 24.0, Armonk, NY; IBM Corp and Excel 2016, Microsoft Corporation. Statistical analyses were approved by a medical statistician.

### Ethical Considerations

The study procedures were performed in accordance with the revised Helsinki Declaration of 1964 and the Good Clinical Practice guidelines. The study was approved by the Regional Ethical Review Board, with registration numbers 2010/49 and 2014/219. The data was coded and deidentified. The included infants were registered according to the regional quality requirements, with number 01481271007173. The database used in this study encrypted all personal identifiers to protect personal privacy. Thus, this study was exempt from requiring patient/family informed consent.

## Results

During the study period, 173 infants underwent the LAG procedure. Data for 27 patients were considered incomplete and were therefore excluded from the study. The missing information was due to lack of a surgery date or information in the patients' electronic medical records, or the patients had their follow-up in other counties. Another five patients underwent reoperation within 30 days of the initial LAG and were therefore excluded from the study. Thus, data from the remaining 141 infants were analyzed.

The median age at surgery was 10 months (range: 1–24) and the frequency of clinically suspected wound infection was 27% (38/141). The demographic data, comprised of age, gender, weight, length, and the most important underlying diagnosis for each infant, are summarized in Table 1 . The median time interval from surgery to clinical infection was 14 days (range: 4–30 days).

**Table 1:** Demographic characteristics of infants < two years of age who underwent LAG from 2010 to 2017

Number of infants included in the study, *n*	141
Number of children with infection	38 (27%)
Girls/boys	68 (48%)/73 (52%)
Age at operation, months (range)	10 (1–24)
Weight, kg (range)	7.1 (3.2–11.30)
Z-score a for weight (range)	–1.5 (–4––1.8)
Diagnoses	
•Neurological disease	72 (51%)
•Cardiac malformations	18 (13%)
•Metabolic disease	14 (10%)
•Syndrome	16 (11%)
•Respiratory insufficiency	10 (7%)
•Gastrointestinal malformations	7 (5%)
•Malignancy	4 (3%)

Abbreviation: LAG, laparoscopic assisted gastrostomy.

The numbers are presented as the absolute number and percentage of patients, *n* = 141 (100%) and as median (min-max).

a Z-scores are calculated as (actual weight–mean weight)/standard deviation according to the national standardized weight curves.

Table 2 summarizes the frequency, culture, and treatment for wound infections. All wound infections occurred at the gastrostomy tube site, with none occurring at the optic port site. There was no spillage of the stomach contents into the abdominal cavity or in the wound and no other perioperative complications were noted in this cohort of children. Among the 38 patients with clinically suspected infections, 30 (79%) were treated with antibiotics. Of the 26 patients who had a positive bacterial culture, 23 (61%) were treated with antibiotics.

**Table 2:** Results of bacterial culture and antibiotic treatment in the 38 children with a clinically diagnosed wound infection after LAG

	Bacterial culture within 30 days after a gastrostomy procedure			Total
Antibiotic treatment < 30 days	Positive	Negative	No culture	
Yes	23 (61%)	2 (5%)	5 (13%)	30 (79%)
No	3 a (8%)	4 (10%)	0 (0%)	7 (18%)
Yes (other reason)	0	1 (3%)	0	1 (3%)
**Total**	26 (69%)	7 (18%)	5 (13%)	38 (100%)

Values presented as the absolute number and percentage of patients, 38 (100%)

a Cultures from these three patients culture grew Staphylococcus aureus, Klebsiella pneumoniae, and mixed gram-negative flora.

The microorganisms cultured from the gastrostomy during the first 30 postoperative days are summarized in Table 3 . Many of the bacteria identified were typical skin bacteria, but 16/37 (43%) originated from gastrointestinal or respiratory flora. The antibiotics used for treatment of LAG infection are summarized in Table 4 and the findings indicate arbitrary use of antibiotics.

**Table 3:** Bacteria/fungi found in gastrostomy wound cultures from 23 of 26 laparoscopic-assisted gastrostomy patients with clinical wound infections within 30 postoperative days

Bacteria/fungi	Infants
**Skin bacteria**	
• *Staphylococcus aureus*	17
•Other skin flora	3
• *Streptococcus pyogenes*	1
**Intestinal bacteria**	
•Mixed gram-negative flora	3
• *Enterococcus faecalis*	3
• *Escherichia coli*	1
• *Serratia marcescens*	1
•Gram negative bacilli	1
**Respiratory bacteria**	
• *Klebsiella pneumoniae*	2
• *Haemophilus influenzae*	2
**Fungi**	
• *Candida albicans*	2
**Other**	
•Mixed flora	1

**Table 4:** Type of antibiotics used to treat infections in 30 patients within the first 30 postoperative days after laparoscopic-assisted gastrostomy

Type of antibiotics	Number
Flucloxacillin (Heracillin)	15
Cefadroxil	4
Sulfametoxazol/Trimetoprima (Bactrim)	2
Cefotaxime	1
Claforan	1
Ceftibuten	1
Clindamycin (Dalacin)	1
Hydrogen peroxide (Microcid)	1
Antibiotics for other reasons	1
Nonspecified antibiotics	4

Logistic regression analysis did not find gender, age at operation, weight at operation, or diagnosis to be independent risk factors for wound infection ( Table 5 ).

**Table 5:** Results of logistic regression analysis of possible risk factors for 30-day postoperative wound infection after laparoscopy-assisted gastrostomy in children < 2 years

Wound infection vs	*p* -Value	Odds ratio	95% CI	
			Lower—Upper
Sex	0.270	0.628	0.274–1.436
Age at operation, months	0.295	0.960	0.889–1.036
Diagnoses	0.337	0.453	0.044–4.908
Weight, kg	0.052	1.354	1.026–1.788

Abbreviation: CI, confidence interval.

## Discussion

The results revealed a clinical wound infection rate of 27% after LAG in infants, with a broad mix of bacteria species in the cultures. Antibiotic treatment was prescribed for 79% of those with positive cultures. Neither gender, age at operation nor diagnosis was found to be risk factors for wound infection. The new knowledge gained from this study is the frequency of wound infections and the bacteriological spectrum found following the LAG intervention in infants using a technique practiced at our center including the application of subcutaneous (SC) stay sutures.

A literature search uncovered no earlier reports on bacterial growth after LAG for comparison. To the best of our knowledge, this is the first report on postoperative infections after LAG in infants (younger than 2 years of age). Searching the electronic database for “gastrostomy AND infant AND laparoscopic-assisted AND wound infection” revealed no literature on the method of operation used at our center. However, the frequency of infection matches the results of previous reports from other types of gastrostomy operations with reported values of 15 to 28%. 2 3 4

Extending the literature search to other methods for performing the gastrostomy intervention revealed a report on the rate of infections following percutaneous endoscopic gastrostomy in infants and children. A retrospective, single-center study that comprised 129 children aged up to 18 years with a median age of 2.9 years reported peristomal infections as the most frequent complication in 10%. 18 In a report from nine tertiary centers on prospective clinical data collection from 239 children with a mean age of 6 years, percutaneous endoscopic gastrostomy tube implantation was conducted. The cumulative incidence of complications was 47.7% at 24 months. No risk factors were identified in association with complications during the first tube replacement. 19

In a report on gastrostomy performed with fundoplication in 100 patients, the authors devised a laparoscopic triangle fixation technique for gastrostomy and interestingly noted a lower complication rate, especially for infection. 20 In another study on persistent gastrostomy site infection (PGSI) after Nissen fundoplication and gastrostomy in 40 patients 1 to 49 years old (median, 11 years), the authors found that PGSI correlated with the perioperative management of positive pressure and with increased intragastric pressure resulting from pyloric obstruction, which is caused by the aberrant distribution of the gastrostomy tube to the pyloric side. 21

The importance of the suture technique for postoperative surgical site infections was studied in a retrospective cohort analysis including 682 pediatric patients younger than 18 years of age who underwent LG placement. The patients were grouped according to the suture techniques used to secure the stomach to the anterior abdominal wall: temporary or SC absorbable sutures. The postoperative outcomes at 30 days showed no significant differences in the development of minor complications including surgical site infections between SC and temporary suture techniques. 22

The use of U-stitch LG 4 for the placement of balloon gastrostomy for pediatric patients, in which the stay sutures are placed in SC tissue, may lead to suture knot abscess formation. 23 Twenty-seven consecutive patients were evaluated, whereby the stay suture knots were positioned within the gastrostomy tract instead of the SC tissue in an attempt to minimize suture knot abscess formation. The results showed that the SC placement of stay sutures within the open gastrostomy tract rather than within closed SC tissue may minimize suture knot abscess formation. 23

The original method for securing a LG involves the placement of two monofilament transabdominal (TA) sutures, to be removed after a short interval of 5 days. 24 The U-stitch LG technique employs an absorbable suture tunneled SC. 4 In a retrospective review of 740 patients who underwent LG placement, dividing the patients into two cohorts according to the securing stitch type (TA and SC) revealed a significantly higher rate (19%) of infectious complications in the SC stitch patients. 25

In summarized reports regarding other methods of performing gastrostomy, the patients were older, and the frequency of infection was similar to that in our previous reports when we did not apply the detailed criteria for infectious complications used in the present report. 16

The endpoint in this study was infectious complications within the first 30 postoperative days, whereas clear endpoints were not always defined in the other studies.

The median interval from surgery until the health-care providers noted the wound infection was 14 postoperative days, which is later than our expectation. This delay could be due to the parents delaying hospital visitation or may indicate that the infection was prevented in the early postoperative period by local treatment using Hibiscrub. If the latter is correct, the delay may indicate that prolonged local treatment could be effective at preventing later infections. Another possible reason for late occurring infections is the presence of foreign material, specifically the gastrostomy button in the gastrostomy site, to which various kinds of bacteria might adhere to over time.

The surgical methods used for gastrostomy differ across various centers. 18 19 20 21 22 23 24 However, with all techniques, a foreign body is left in the wound with either stitches or T-fasteners used to anchor the stomach to the anterior abdominal wall. In addition, the infant's clinical situation, periods of cardiac insufficiency, and various phases of treatment with cytostatic drugs may influence healing. The situation might be further complicated by the intraabdominal and SC resorbable U-stitch suture (Vicryl or Biosyn). This was used in our study to suture around the gastrostomy opening to anchor the stomach to the anterior abdominal wall 2 3 4 according to the original procedure described by Stamm. 26 Since bacteria did not grow in all bacterial cultures collected, we cannot exclude the possibility that normal hydrolysis of the resorbable suture was mistaken for an infection because it can appear as redness and irritation around the gastrostomy.

The frequency of postoperative wound infections after LAG has been reported to vary according to the underlying diagnoses. 16 This report was not supported by the findings in this study. The number of infections documented in this report may, however, be too small to reveal significant correlations between a specific diagnosis and the risk of postoperative wound infection. Previous studies did not demonstrate the influence of cytostatic drugs on wound healing or infectious complications after LAG 14 and this was not reviewed in our study.

Many of the bacteria cultured from the gastrostomy wound were bacterial skin flora. Because skin flora is expected in surgical skin wounds, antibiotic prophylaxis was administered before LAG, as described in the methods section, to prevent infection. However, since most infections were indeed due to skin bacteria, the effect of the antibiotic prophylaxis could be questioned, and other preventive measures may be warranted. Respiratory and intestinal bacteria that grew in some cultures are not expected to grow in the gastrostomy surgical wound and could therefore be opportunistic bacteria or contaminants. Further, some of the respiratory and intestinal bacteria found in some bacterial cultures do not usually cause wound infections and their role in the development of infection is unclear. These bacteria probably require treatment with antibiotics because they may potentially cause serious infections. Local treatment without antibiotics may play a role in replacing antibiotic prophylaxis or even medical treatment for local infections.

Several factors influenced the outcome of the present study. These include the age and body weight of the patients. Although all the included children were lesser than 2 years of age, a substantial difference was noted between the youngest and the oldest in the cohort. These differences included the age, which ranged from 1 to 24 months, and the body weight of the patients, which ranged from 3 to 11 kg. Furthermore, the included children had several diagnoses that may have affected them in different ways during the operative gastrostomy intervention, thereby influencing the outcome in terms of postoperative infections. The diagnosis could be divided into seven categories, with further variations within each category. We know from previous reports that children with cardiac anomalies and pulmonary insufficiency are more often affected by infectious complications than children with neurological dysfunctions. 16

Our study had several limitations. The 141 included infants, all lesser than 2 years in age, represent all infants operated on at a single tertiary center during a period of 8 years by the same group of surgeons. The small sample size is a cause of bias. The greatest limitation of this study is its retrospective nature, which introduces bias to the results. Because the data, which was collected from a database, was not documented intentionally for this study, the protocols and data collection were not uniform, which limited the information available. Additionally, the data used for risk analysis may be too small to draw any conclusions regarding independent risk factors for a postoperative wound infection after LAG. Furthermore, the included children had several diagnoses and there were few children in each diagnosis group, making it impossible to adjust for all possible confounding factors. The significant clinical heterogeneity influenced our conclusions. Furthermore, there was a lack of uniform criteria for reporting the outcomes and we assumed that the criteria were similar enough to be assessed together. Therefore, future studies with complete data and uniform criteria are required to ensure reliable results. Nevertheless, the findings of this study provided valuable and up-to-date information in this field. The level of evidence can be increased with a prospective study.

There was potential bias in the retrospective collection of data, including selection bias. Furthermore, performance bias cannot be excluded due to the binding of personnel and detection bias may be present due to the binding of the outcome assessment. Incomplete outcome data (attrition bias), selective reporting (reporting bias), and publication bias are likewise possible. However, the strength of the design is that it did not influence the treatment of the patients, who were all treated in accordance with local standards.

Increased knowledge of complications after LAG can help inform both parents and health-care professionals about the expected postoperative outcomes. This information might also improve compliance with antibiotic treatment protocols and help minimize unnecessary antibiotic treatments in the future. Thus, the data collected during this work can serve as a benchmark for future clinical works as well as a foundation for further scientific research.

This knowledge can be applied in routine clinical practice when discussing the method of choice for performing gastrostomy in children. Furthermore, knowledge regarding postoperative infectious complications can be of use when deciding on the use of antibiotics and the timing for postoperative control and clinical follow-up of the patients. To apply the knowledge obtained from research in clinician practice, one must consider local context, policy, and habits. Thus, the results of our study can be used as a benchmark for further attempts in improving the postoperative results after LAG tube placement. This can be implemented by using some of the suggestions for improvement already found in the literature 20 22 23 27 28 29 30 31 32 or by new interventions.

## Conclusion

This study revealed a 27% rate of wound infection after LAG despite the use of antibiotic prophylaxis and local antibacterial treatments and 79% of these infants were treated with antibiotics. No independent risk factor for wound infection was identified. Since antibiotic resistance is threatening, postoperative treatment after gastrostomy placement should be expected, and future studies on local hygienic treatment are needed.
